# First Experience With Extracorporeal Cytokine Adsorption Therapy After Lung Transplantation

**DOI:** 10.3389/ti.2022.10319

**Published:** 2022-03-21

**Authors:** Marine Peyneau, Luc de Chaisemartin, Dorothée Faille, Jonathan Messika, Hervé Mal, Yves Castier, Pierre Mordant, José Luis Carrasco, Sébastien Tanaka, Brice Lortat Jacob, Paola Ferrari, Xavier Arrault, Nadine Ajzenberg, Sylvie Chollet-Martin, Philippe Montravers, Alexy Tran-Dinh

**Affiliations:** ^1^ Autoimmunity and Hypersensitivity Laboratory, AP-HP, Hôpital Bichat-Claude-Bernard, Paris, France; ^2^ Faculty of Pharmacy, INSERM UMR 996, Inflammation, Microbiome and Immunosurveillance, Université Paris-Saclay, Châtenay-Malabry, France; ^3^ Hematology Laboratory, AP-HP, Hôpital Bichat Claude Bernard, Paris, France; ^4^ Laboratory for Vascular Translational Science, INSERM UMR 1148, Université de Paris, Paris, France; ^5^ INSERM UMR 1152 PHERE, Université de Paris, Paris, France; ^6^ Pneumologie B et Transplantation Pulmonaire, AP-HP, Hôpital Bichat-Claude-Bernard, Paris, France; ^7^ Paris Transplant Group, Paris, France; ^8^ Service de Chirurgie Vasculaire, Thoracique et Transplantation Pulmonaire, AP-HP, Hôpital Bichat Claude Bernard, Paris, France; ^9^ Département d’Anesthésie-Réanimation, AP-HP, Hôpital Bichat-Claude Bernard, Paris, France; ^10^ INSERM UMR 1188 DéTROI, Université de la Réunion, Saint-Denis de la Réunion, France; ^11^ Service de Pharmacie, AP-HP, Hôpital Bichat Claude Bernard, Paris, France

**Keywords:** lung transplantation, hemoadsorption, cytokine, Cytosorb, inflammation

Dear Editors,

Lung transplantation (LT) is accompanied by pro-inflammatory cytokine release, which correlates with the graft outcome (1–3). Extracorporeal cytokine adsorption therapy (ECAT) by Cytosorb® (CytoSorbents Corporation, Monmouth Junction, United States), a porous polymer beads adsorption cartridge, removes hydrophobic substances of molecular weight ≤60 kDa from the blood. ECAT is a promising therapy in hyperinflammatory situations (4–8), but has never been evaluated in LT. We evaluate for the first time ECAT on both circulating and membrane phagocyte-expressed inflammation biomarkers in the postoperative course of LT.

We conducted a prospective study at Bichat-Claude Bernard Hospital (Paris, France). Consecutive patients undergoing LT and admitted to the intensive care unit (ICU) postoperatively with extracorporeal membrane oxygenation (ECMO) were assessed. Cytosorb® cartridge was integrated into a bypass of the ECMO circuit at ICU admission. ECAT was performed during 24 h with the same cartridge. Blood samples were collected before cartridge placement (T0), after 24 h of ECAT (T1), and 24 h after cartridge removal (T2). We studied the evolution of membrane activation markers of neutrophils (CD66b and CD11b) and monocytes (CD14 and HLA-DR) by flow cytometry (Becton-Dickinson, FACS Lyric), the quantification of plasma levels of IL-6 and IL-8 by Luminex assay (Procartaplex®, Thermofisher) and L-lactate (Radiometer ABL90), and coagulation factors (factors II, V, VII, X, C protein, antithrombin III, and fibrinogen. Clinical data and outcomes are expressed in median (IQR). The study was approved by the French National Ethics Committee “Comité de Protection des Personnes Sud-Est II” (2017-A02625-48).

Six patients were transplanted for fibrosis (*n* = 4), chronic obstructive pulmonary disease (COPD) (*n* = 1) and silicosis (*n* = 1). At T2, neutrophil activation markers CD66b and CD11b expressions were significantly decreased as well as L-lactate levels (Figure 1). A downward trend was observed for monocyte activation markers (CD14 and HLA-DR), IL-6 and IL-8. No rebound effect was observed for any of these markers 24 h after cartridge removal. Coagulation markers were not altered. However, we observed one case of cartridge clotting after 12 h of treatment, without any consequences on the ECMO circuit. At T0, T1 and T2, norepinephrine doses were 0.75 (0.3–1.1), 0.25 (0.04–0.58) and 0.25 (0.03–1.15) μg/kg/min and PaO_2_/FiO_2_ ratio were 77 (74–118), 93 (88–107) and 79 (72–98) mmHg, respectively. Compared with a “control” cohort of 27 transplant patients over the same study period, the ICU length of stay and in hospital were longer for patients with ECAT, respectively of 64 (46–69) vs 41 (33–53) and 121 (82–146) vs 45 (38–63) days. However, at 1 year after LT, patients with ECAT were all alive, whereas the survival rate for patients in the “control” cohort without ECAT was 70.4%.

**FIGURE 1:**
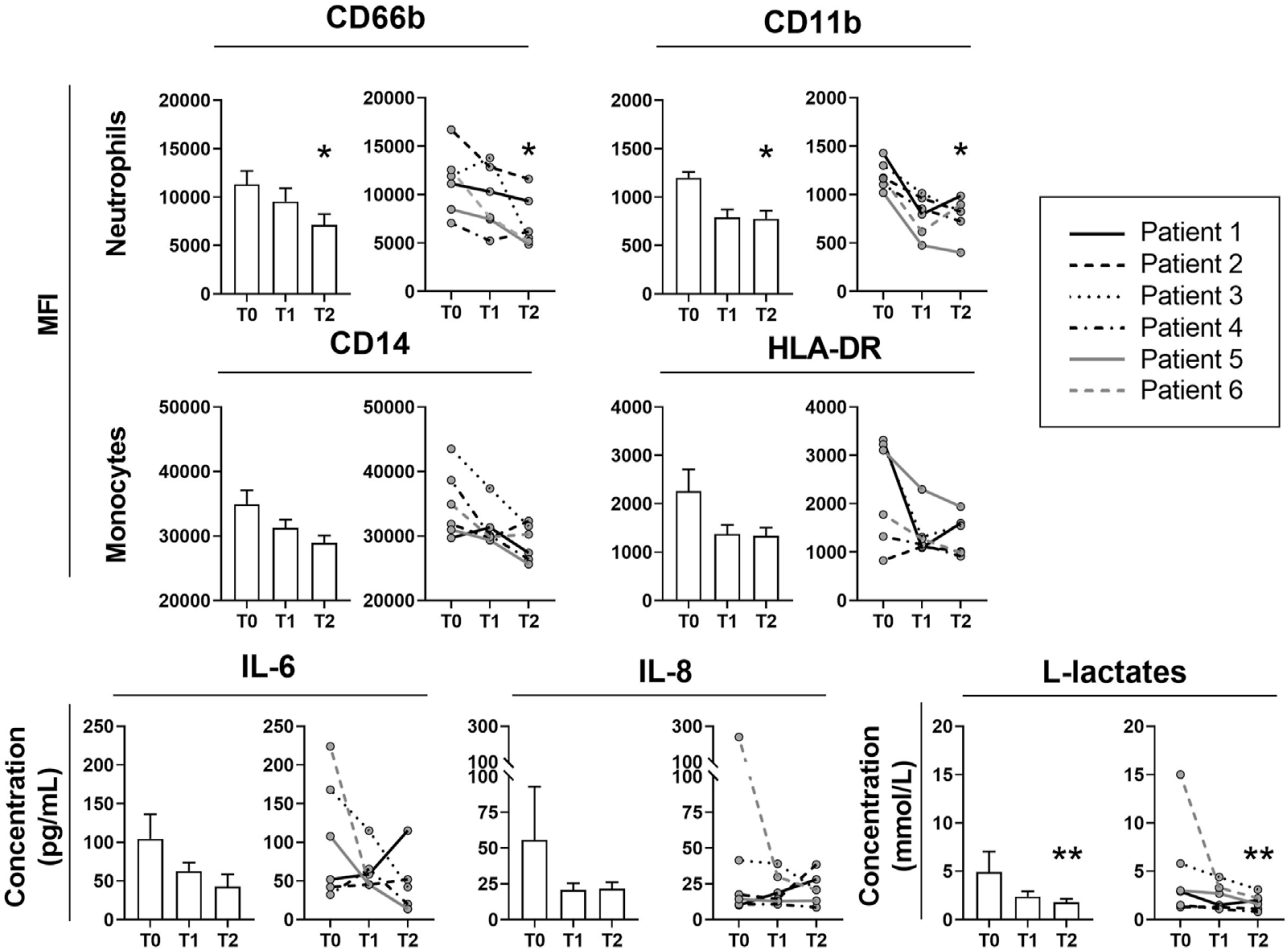
Evolution of neutrophil and monocyte membrane activation markers, IL-6, IL-8 and L-lactate during extracorporal cytokine adsorption therapy by Cytosorb®. Activation membrane markers of neutrophils (CD66b and CD11b) and monocytes (CD14 and HLA-DR) were assessed by flow cytometry before cartridge placement (T0), after 24 h of ECAT (T1), and 24 h after cartridge removal (T2) and are expressed as mean fluorescence intensity (MFI). IL-6 and IL-8 were quantified by Luminex assay and L-lactates were assessed with Radiometer ABL90 Flex and concentrations are expressed in pg/ml and mmol/L respectively. Data are expressed in histograms (left) as the mean ± SEM or as individual values (right). Friedmann’s test and Dunn’s *post-hoc* tests are represented. *p < 0.05 **p < 0.01, as compared to T0.

We present the first pilot study on the feasibility and efficacy of ECAT after LT. The decrease in neutrophil and monocyte activation markers has never been reported before and suggests a possible indirect immunomodulatory effect of ECAT on phagocyte activation. The decreased plasma IL-6 and IL-8 concentrations was not significant. However, the three patients with elevated IL-6 and/or IL-8 levels at T0 experienced a dramatic decrease at T1. Cytosorb® appears to be a safe and promising device to fight post-LT inflammation, and should be re-evaluated in a larger study.

## Data Availability

The raw data supporting the conclusion of this article will be made available by the authors, without undue reservation.

## Abbreviations

COPD, chronic obstructive pulmonary disease; ECAT, extracorporeal cytokine adsorption therapy; ECMO, extracorporeal membrane oxygenation; ICU, intensive care unit; LT, lung transplantation.
